# Supplementary material for: Inflammation-Related Biomarkers Are Associated with Heart Failure Severity and Poor Clinical Outcomes in Patients with Non-Ischemic Dilated Cardiomyopathy
Source: Life (Basel). 2021 Sep 24;11(10):1006. doi: 10.3390/life11101006 (PMC8540264; doi:10.3390/life11101006)
Supplement: Supplementary file 1 [file life-11-01006-s001.zip › life-1375120-supplementary.pdf]

life

MDPI

Table S1. Cut-off values identified by ROC analysis of inflammation-related biomarkers for predicting adverse cardiac events.

	Cut-off	Specificity, %	Sensitivity, %	AUC (95% CI)
WBC × 10^9/L	10.66	32	88	0.56 (0.39–0.72)
hs-CRP, mg/L	4.62	65	65	0.61 (0.44–0.77)
IL-6, pg/mL	4.53	83	64	0.77 (0.64–0.89)
TNF-α, pg/mL	7.81	53	80	0.65 (0.50–0.79)
Adiponectin, μg/mL	17.14	73	60	0.70 (0.55–0.73)
suPAR, ng/mg of protein	0.85	80	35	0.51 (0.34–0.69)
CD3+, cells/mm^2	13	75	41	0.59 (0.43–0.74)
CD45ro+, cells/mm^2	11.5	89	26	0.53 (0.37–0.68)
CD68+, cells/mm^2	9	96	11	0.51 (0.36–0.66)
CD54+, cells/mm^2	2.5	93	7	0.43 (0.29–0.56)
CD4+, cells/mm^2	3.5	61	56	0.53 (0.37–0.69)
HLA-DR+, cells/mm^2	5.5	68	41	0.53 (0.38–0.68)

AUC–area under the curve, hs-CRP–high-sensitivity C-reactive protein, CI–confidence interval, IL-6–interleukin 6, suPAR–soluble urokinase-type plasminogen activator receptor, TNF-α–tumor necrosis factor α, WBC–white blood cell.
